# A Review on Current Aspects of Curcumin-Based Effects in Relation to Neurodegenerative, Neuroinflammatory and Cerebrovascular Diseases

**DOI:** 10.3390/molecules30010043

**Published:** 2024-12-26

**Authors:** Claudia-Andreea Moldoveanu, Maria Tomoaia-Cotisel, Alexandra Sevastre-Berghian, Gheorghe Tomoaia, Aurora Mocanu, Csaba Pal-Racz, Vlad-Alexandru Toma, Ioana Roman, Madalina-Anca Ujica, Lucian-Cristian Pop

**Affiliations:** 1 Department of Molecular Biology and Biotechnology, Babeș-Bolyai University, Clinicilor St., RO-400371 Cluj-Napoca, Romania; cmoldoveanu4@gmail.com; 2 Department of Experimental Biology and Biochemistry, Institute of Biological Research from Cluj-Napoca, a Branch of NIRDBS Bucharest, 48 Republicii St., RO-400015 Cluj-Napoca, Romania; ioana.roman@icbcluj.ro; 3 Research Center of Excellence in Physical Chemistry, Faculty of Chemistry and Chemical Engineering, “Babes-Bolyai University”, 11 Arany Janos St., RO-400028 Cluj-Napoca, Romania or maria.tomoaia@ubbcluj.ro (M.T.-C.); mocanu.aurora@gmail.com (A.M.); csaba.racz@ubbcluj.ro (C.P.-R.); madalina.ujica@ubbcluj.ro (M.-A.U.); 4 Academy of Romanian Scientists, 3 Ilfov St., RO-050044 Bucharest, Romania; tomoaia2000@yahoo.com; 5 Department of Physiology, Faculty of Medicine, “Iuliu Hațieganu” University of Medicine and Pharmacy, 1 Clinicilor St., RO-400006 Cluj-Napoca, Romania; berghian.alexandra@umfcluj.ro; 6 Department of Orthopedics and Traumatology, “Iuliu Hațieganu” University of Medicine and Pharmacy, 47 Gen. Traian Moșoiu St., RO-400132 Cluj-Napoca, Romania; 7 Centre for Systems Biology, Biodiversity and Bioresources “3B”, Babeș-Bolyai University, 44 Republicii St., RO-400347 Cluj-Napoca, Romania

**Keywords:** curcumin, bioavailability, formulations blood–brain barrier, cerebrovascular diseases, benefits and limiting effects

## Abstract

Curcumin is among the most well-studied natural substances, known for its biological actions within the central nervous system, its antioxidant and anti-inflammatory properties, and human health benefits. However, challenges persist in effectively utilising curcumin, addressing its metabolism and passage through the blood–brain barrier (BBB) in therapies targeting cerebrovascular diseases. Current challenges in curcumin’s applications revolve around its effects within neoplastic tissues alongside the development of intelligent formulations to enhance its bioavailability. Formulations have been discovered including curcumin’s complexes with brain-derived phospholipids and proteins, or its liposomal encapsulation. These novel strategies aim to improve curcumin’s bioavailability and stability, and its capability to cross the BBB, thereby potentially enhancing its efficacy in treating cerebrovascular diseases. In summary, this review provides a comprehensive overview of molecular pathways involved in interactions of curcumin and its metabolites, and brain vascular homeostasis. This review explores cellular and molecular current aspects, of curcumin-based effects with an emphasis on curcumin’s metabolism and its impact on pathological conditions, such as neurodegenerative diseases, schizophrenia, and cerebral angiopathy. It also highlights the limitations posed by curcumin’s poor bioavailability and discusses ongoing efforts to surpass these impediments to harness the full therapeutic potential of curcumin in neurological disorders.

## 1. Introduction

Cerebrovascular illnesses are a leading source of illness and death worldwide, encompassing conditions such as ischemic/haemorrhagic stroke and also transient ischemic attack. The incidence and prevalence vary across different populations but generally increase with age. Ischemic stroke is more common than haemorrhagic stroke, accounting for about 85% of all strokes. However, the detrimental effects of haemorrhage are more prominent than ischemia-reperfusion conditions due to haemoglobin toxicity, high redox imbalance, inflammation, and a high risk of thromboembolism [1,2].

Several risk factors lead to the emergence of cerebrovascular diseases. These include modifiable causes like hypertension, smoking, diabetes mellitus, hyperlipidemia, obesity, a poor diet, and a lack of exercise, while non-modifiable risk factors include age or gender. Men are at higher risk at younger ages, while women present an increased risk of stroke after menopause [3]. Prophylactic strategies for stroke and cerebrovascular diseases, such as microangiopathy, neurodegeneration, and neuropathy, are restricted to maintaining a healthy lifestyle, avoiding smoking and alcohol, practising good sleep hygiene, and consuming a healthy diet [4].

Since the early 2000s, there has been an increase in interest in scientific clinical research to examine the efficacy, safety, and mechanisms of action of plant-based therapies, including curcumin, resveratrol, alkaloids with antitumoural properties, or plant extracts for metabolic diseases [5,6]. Research has looked into the potential therapeutic benefits of numerous plant extracts and isolated compounds for conditions ranging from chronic diseases like cardiovascular disorders, diabetes, and cancer to common ailments like colds, digestive issues, and anxiety [7,8].

The relationship between curcumin and cerebrovascular diseases has been well established through various studies highlighting its effects on several cerebral conditions. However, the mechanisms underlying curcumin’s ability to cross the blood–brain barrier (BBB), the experimental models used to study it, and its interaction with endothelial cells remain cutting-edge topics in biopharmacy. Previous research has shown that insoluble antioxidants like curcumin significantly enhance endothelial function by improving nitric oxide (NO) bioavailability. Curcumin prevents NO degradation by reactive oxygen species (ROS), thereby promoting vasodilation and enhancing blood flow. Moreover, these antioxidants inhibit the oxidation of low-density lipoproteins (LDL), a crucial step in atherosclerosis development. By reducing LDL oxidation, curcumin minimises plaque formation and vascular occlusion. Additionally, curcumin helps stabilise vascular structures by shielding them from oxidative damage, preserving vascular integrity, and reducing the risk of aneurysms. It also influences lipid metabolism by modulating allosteric enzymes, which lowers harmful lipid levels and mitigates cardiovascular risk. Together, these evidence-based findings underscore the significant potential of curcumin in cerebrovascular health and disease, paving the way for deeper exploration into its molecular mechanisms and therapeutic applications.

This review offers an original and comprehensive overview of the present level of understanding related to curcumin-based therapies, emphasising their potential as valuable additions to conventional medicine. It emphasises how crucial it is to conduct more studies to clarify their mechanisms of action, safety profiles, limitations, and clinical efficacy in cerebral and cerebrovascular diseases. These diseases include ischemic and haemorrhagic stroke, diabetic microangiopathy, neurodegenerative pathologies; for example, Alzheimer’s and Parkinson’s, schizophrenia, diabetic neuropathy, and metal-induced neurotoxicity.

Furthermore, the review addresses the gap between curcumin actions in normal and neoplastic cells. It also dedicates a distinct chapter to discussing prospects and challenges for curcumin-based therapies.

## 2. Curcumin’s Metabolism and Bioavailability Related to Brain Physiology

### 2.1. Curcumin Metabolism

Curcumin, the principal polyphenolic compound derived from *Curcuma longa* L. (turmeric), exhibits slight solubility in water but solubility in ethanol and acetone. Various review articles have summarised curcumin metabolism [9], involving processes such as oxidation, cleavage, reduction, and conjugation [10,11]. Studies on rodents have also looked into curcumin’s distribution, metabolism, excretion, and absorption [12,13]. Curcumin is metabolised, mostly in the intestine and liver. The major metabolic pathways include reduction, conjugation, and microbial metabolism. It is crucial to remember that the metabolites of curcumin, particularly the conjugated forms, exhibit reduced biological activity compared to the parent compound. This reduction in bioactivity may limit the therapeutic efficacy of curcumin, especially considering its poor bioavailability [14,15,16,17].

Efforts to intensify curcumin’s bioavailability include the growth of novel systems for delivery such as nanoparticle formulations, liposomes, and micelles, which aim to improve its solubility, stability, and absorption. Additionally, strategies to inhibit metabolic enzymes or enhance intestinal permeability are being explored to go beyond the restrictions imposed by curcumin metabolism. To extend the amount of time curcumin remains in the body, different formulation approaches were used. For instance, complexes between curcumin and phospholipid can prolong the retaining of curcumin in rodent serum [18]. According to other studies, the mean residence time of curcumin greatly increased following micellar formulation [19,20]. In 2010, Mohanty and Sahoo [19] discovered that encapsulating curcumin in glycerol monooleate lengthens its half-life. Katz and Tomoaia-Cotisel [21] showed in 1999 that delivery systems based on polymers of synthesis are advantageous for hydrophobic substances and increase the bioavailability of medicines that are not highly water soluble.

Site-specific biomolecular complexes that contain a therapeutic, preventative, and diagnostic agent—also known as a physiologically active molecule—are described in one of the patents pertaining to the curcumin–BBB interaction. Omega-3 fatty acids, like alpha-linolenic, eicosapentaenoic, or docosahexaenoic acid, and their derivatives are included in the recommended therapeutic complexes or conjugates [21]. The transport of a biologically active ingredient (like curcumin) to the brain’s glial tissue, along with cortical, cholinergic, and adrenergic neurons, is made possible by these complexes. Research has indicated that the way curcumin is administered can affect its bioavailability. Numerous attempts with notable improvements in plasma bioavailability have also documented the benefits of oral delivery. When administered intravenously, certain nanoformulations have been shown to improve tissue distribution and absorption [22]. Using a special combination of proteins isolated from fenugreek seeds and soluble dietary fibre (galactomannans), scientists recently revealed a novel formulation of curcumin called curcuma-galactomannosides (CGM), which has improved bioavailability in both humans and animals [23].

In this study, curcuminoids and their metabolites were quantified using UPLC-ESI-MS/MS. The method involved tandem mass spectrometry (MS/MS) in MRM mode, with electrospray ionisation (ESI) operating under positive and negative ion polarities, allowing separation, detection, and confirmation of curcuminoids from various rat biomatrices. It was hypothesised that achieving significant levels of free curcuminoids in brain tissues depends on maintaining high plasma levels for a sufficient duration to enhance BBB permeability. Using Wistar rats and UPLC-ESI-MS/MS, the study confirmed CGM’s bioavailability through tissue distribution analysis rather than plasma metabolite quantification. The results indicate that biologically active curcuminoids can reach target brain tissues upon oral administration of CGM, enabling their pharmacodynamic activity. CGM’s solvent-free preparation method also enhances its value as a potential dietary supplement or functional food [23].

According to earlier research, CGM has strong anti-inflammatory properties that stop the production of reactive oxygen species and proinflammatory cytokines in a variety of in vivo neurotoxic models [24,25,26].

As previously stated, substantial progress has been achieved in the creation of medicine transport systems that use liposomes, nanoparticles, and microemulsions as curcumin carriers in recent years. Surface modification of nanocarriers has enabled them to exploit receptors overexpressed at the BBB, facilitating efficient penetration and targeted delivery of curcumin to precise sites inside the brain. This targeted delivery method augments the therapeutic efficacy of curcumin for neurological conditions. Moreover, the choice of administration route plays a crucial role in achieving optimal distribution of brain drugs. Thus, the advanced drug delivery systems and the understanding of optimal administration routes are vital for harnessing curcumin’s whole medicinal potential, particularly in neurological disorders.

Countless methodologies have been proposed to enhance BBB penetration and effective drug discharge outside of the brain, particularly for targeting glioblastoma multiforme and other neurological disorders:

1. Encapsulation in surface-modified poly (amidoamine) (PAMAM) dendrimers: Curcumin has been encapsulated in fourth-generation PAMAM dendrimers, which are surface-modified to improve BBB penetration. In vitro studies employing therapeutic quantities of encapsulated curcumin demonstrated significant reductions in the viability of glioblastoma cells from different species [26].
2. Solid dispersion: Solid dispersion involves dispersing drugs in highly soluble carriers to enhance solubilisation, particularly for insoluble drugs like curcumin. Methods like hot melt extrusion, solvent evaporation, and spray drying are utilised to achieve fine-grain dispersion and improve solubility [27,28].
3. Ultrasound-induced BBB opening: This is a non-invasive technique that avoids implants, making them attractive to patients. It enables targeted drug delivery to specific, widely distributed brain regions, with adjustable parameters like ultrasound settings and microbubble dosing. The method has been tested in various species, including rodents, large animals, and humans, targeting areas such as the hippocampus, striatum, and tumour tissues. While safe for repeated use, FUS is particularly suitable for therapies with infrequent dosing, like gene therapy, to reduce chronic exposure risks [29]. One application involves delivering curcumin to treat Parkinson’s disease (PD) in mice, using lipid-PLGA nanobubbles and low-intensity ultrasound to enhance effectiveness [30].
4. Carrier-molecule conjugates: Another method proposes enhancing the ability of neurologically active compounds to penetrate the blood–nerve barrier (BNB) or BBB by administering conjugates comprising the active compound linked to carrier molecules with substantial permeability coefficients across the BNB and BBB [31].

### 2.2. Curcumin’s Bioavailability in the Central Nervous System

Curcumin’s bioavailability within the central nervous system is influenced by the blood–brain barrier (BBB), a protective layer primarily consisting of cerebral microvascular endothelial cells, astrocytes, basal lamina, and pericytes. The BBB’s semi-permeable and selective properties are facilitated by tight connections between capillary endothelial cells, aimed at maintaining stability in the nervous system’s internal environment and preventing the penetration of very large molecules into the brain parenchyma [32,33,34]. This selective barrier hampers the passage of many drugs, peptides, and large molecules, serving as a boundary between the brain and the systemic circulation [35,36]. Only very liposoluble substances with small molecular weight can effectively traverse this barrier and reach the brain [37]. However, the BBB presents a significant challenge in treating neurodegenerative diseases, as it limits drug delivery to the brain. To address this challenge, ongoing research aims to develop innovative strategies and pharmaceutical formulations that enhance BBB penetration, offering potential solutions for improved brain drug delivery.

**Figure 1:**
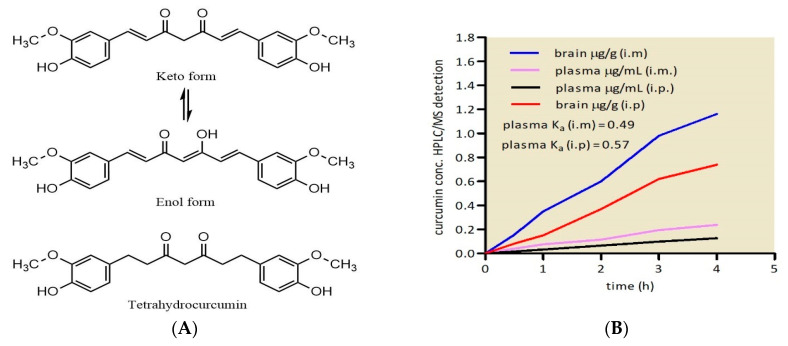
(**A**). Curcumin in its keto and enol forms, and tetrahydrocurcumin (TC) structure. (**B**). The plasma–brain absorption curves for curcumin. The passage from plasma to the brain was influenced by the administration route, with the absorption coefficient Ka being higher in i.p. administration (0.57 μg/h) than in i.m. administration (0.49 μg/h) of curcumin. According to Begum et al. (2008), in mice, tetrahydrocurcumin (TC), a more stable curcumin metabolite, was detected with similar dynamics. After 4 h of intramuscular (i.m.) curcumin administration, plasma TC concentration was 0.971 µg/mL, and brain TC concentration was 2.234 µg/g. In contrast, intraperitoneal (i.p.) administration showed lower TC concentrations, with plasma TC at 0.847 µg/mL and brain TC at 0.765 µg/g. The initial dose of curcumin was 148 µg/kg b.w.; i.m. intramuscular, i.p. intraperitoneal [43].

Several studies have highlighted the critical issue of low curcumin bioavailability following oral intake, hindering the achievement of therapeutic concentrations in the blood [38]. Numerous factors contribute to curcumin’s unfavourable pharmacokinetics, including its extremely poor solubility in water (approximately 11 ng/mL) [39,40], inadequate gut absorption, rapid metabolism, limited distribution, and swift excretion [14,41]. Furthermore, the relation between curcumin brain availability after intramuscular, i.m., and intraperitoneal, i.p., administration is properly described [42]. Accordingly, tetrahydrocurcumin (TC) was detected, in mice, as the more stable curcumin’s metabolite; the curcumin and TC structure is given in Figure 1A. Figure 1B shows the relation between curcumin brain availability after intramuscular and intraperitoneal administration. The calculated absorption half-life for curcumin was 1.41 h after i.m. administration and 1.21 h after i.p. administration. Derived absorption coefficient Ka was calculated as ln (2) divided by absorption t_1/2_. Hence, for i.m. administration, Ka = 0.49 μg/h while Ka for i.p. administration was 0.57 μg/h.

Curcumin coexists in keto and enol forms, and TC might mediate curcumin’s efficacy [44,45]. The curcumin’s passage from plasma to the brain was shown to be influenced by the administration route.

Notably, there are numerous pharmacological effects of curcumin, including anticancer, antioxidant [46], anti-inflammatory, and neurogenic properties [47,48], as well as antibacterial, free radical scavenging, and antidepressant activities [49,50,51]. Among its multifaceted pharmacological effects, the neuroprotective potential of curcumin has garnered significant interest, particularly in traumatic brain damage, spinal cord injury, neuropsychiatric conditions, neurodegenerative illnesses, and epilepsy [52]. Studies suggest that its neuroprotective mechanisms might involve the modulation of neurotransmitters, the hypothalamus–pituitary–adrenal cortex axis, neurotrophic factors’ release, and encouraging nerve regeneration. These mechanisms influence various signalling pathways, enhancing neuronal vitality, differentiation, and ultimately neurological function [53,54,55]. Furthermore, curcumin has shown efficacy in addressing hippocampal dysfunction associated with stroke, trauma, radiation, and neurodegenerative diseases [56]. Its neuroprotective characteristics are ascribed to its anti-amyloid, antioxidant, and anti-inflammatory properties [35]. By preventing oxidation, apoptosis, and inflammation, shielding the BBB, and reestablishing mitochondrial function, curcumin also aids in the healing process following ischemia injury [9,21,57]. Additionally, it reduces nitric oxide concentration, thereby protecting the brain from lipid peroxidation [35,58].

However, curcumin’s low bioavailability in the brain limits its usefulness despite its strong neuroprotective qualities. This limitation arises from factors such as reduced absorption and stability at biological pH, rapid metabolism, systemic removal, and inadequate permeation through the blood–brain barrier [21,23,25]. Curcumin is poorly absorbed when ingested because of its insolubility and gastrointestinal conditions, then, rapid glucuronidation/sulfation of the absorbed portion results in inactive metabolites with low cellular permeability and quick systemic removal [47].

## 3. Curcumin and Neuro-Vascular Pathologies

### 3.1. Curcumin Effects in Brain Ischemia and Stroke

Curcumin has been investigated for its ability to mitigate the pathophysiological processes involved in ischemic stroke and promote neuroprotection through 3 mechanisms:

(i) Anti-inflammatory and antioxidant effects [59]: Curcumin exhibits potent anti-inflammatory characteristics by suppressing the activation of inflammatory pathways and diminishing the production of pro-inflammatory mediators like IL-1β (interleukin-1 beta), IL-6 (interleukin-6), MCAP-1 and TNF-α with a prominent role of the IL-1β in hypoxia-related detrimental effects [60] as well as in the progression of ischemic brain injury, contributing to neuronal damage and exacerbating stroke outcomes. Curcumin’s ability to attenuate neuroinflammation [61] may help mitigate secondary brain injury and promote tissue repair following ischemic stroke by Akt/Nrf2 pathway stimulation, upregulation of the brain-derived neurotrophic factor (BDNF) expression, and suppression of the NAD(P)H: Quinone oxidoreductase 1 (NQO1) induced by brain hypoxia [62,63,64]. By scavenging free radicals and avoiding lipid peroxidation, curcumin also functions as a strong antioxidant [65] and upregulates endogenous antioxidant enzymes like GPx1, GPx4, CAT, and SOD1 [66,67]. By reducing oxidative damage, curcumin may protect neurons from ischemic injury and promote neuronal survival.
(ii) Anti-apoptotic effects: Apoptosis is a prominent feature of ischemic stroke pathology, leading to neuronal loss and tissue damage [68]. Curcumin has been shown to modulate apoptotic pathways by downregulating the expression of pro-apoptotic [69] and upregulating the expression of the anti-apoptotic proteins [70], thereby promoting cell survival and reducing neuronal death and glial activation in ischemic conditions in the daily administration of ~ 2g/kg diet during 2 months [71]. Brain pro-apoptotic factors inhibited by curcumin are caspase-3, Fas and its ligand (FasL), Bax, Bcl2 [72,73] with notable differences as compared to cancer cells or other tissues where the brain anti-apoptotic signalling due to curcumin was observed as pro-apoptotic behaviour like PI2K/Akt inhibition, Fas/FasL upregulation of Bax/Bcl2 increasing [74,75,76]. Moreover, recent studies observed that curcumin inhibited proteasomal degradation and reduced apoptosis [77,78] but these findings must be subjected to more experimental data considering their action in neoplastic processes as a pro-tumoural agent.

The anti-apoptotic effects of curcumin have been extensively studied in various in vivo models of neurodegenerative diseases, including Parkinson’s disease (PD), Alzheimer’s disease (AD), and cerebral ischemia. Its neuroprotective properties are primarily attributed to antioxidant, anti-inflammatory, and anti-apoptotic mechanisms.

In animal models of PD, curcumin has demonstrated significant potential in protecting dopaminergic neurons and mitigating oxidative stress. For instance, it exhibits protective effects against neurotoxins such as 6-hydroxydopamine (6-OHDA) by modulating apoptosis and inflammation-related signalling pathways. Additionally, curcumin enhances neuronal survival and improves behavioural and biochemical outcomes in these models [79].

In AD models, curcumin has been shown to modulate apoptotic pathways by interacting with proteins and genes that regulate cell survival. It effectively reduces pro-inflammatory cytokines and oxidative stress markers, thereby supporting neuronal survival and providing neuroprotection [80].

Moreover, curcumin promotes a shift in microglial activation from a pro-inflammatory (M1) to an anti-inflammatory (M2) state, which reduces apoptosis and facilitates tissue repair. This activity contributes to the preservation of the blood–brain barrier and minimises damage associated with ischemic events [81].

(iii) Neuroprotection against excitotoxicity, resulting from excessive glutamate release and subsequent calcium influx, contributes to neuronal injury in ischemic stroke [82]. Curcumin has been reported to modulate glutamate receptors, prevent calcium influx, and attenuate excitotoxic cell death by regulating excitotoxic signalling pathways [83]. Some authors observed the upregulation influence of curcumin on brain-derived neurotrophic factor after neuron exposure to 10 µM of sodium glutamate which was followed by decreased cell viability and improved cell apoptosis. Curcumin pretreatment of neurons resulted in a dose- and time-dependent reversal of BDNF expression and cell survival. Nevertheless, the survival-promoting impact of curcumin has been abolished when neurons are exposed to a Trk receptor inhibitor, which is known to suppress BDNF activation. Additionally, K252a, a Trk receptor inhibitor, inhibited curcumin’s upregulation of BDNF mRNA and protein. When combined, these findings implied that the BDNF/TrkB signalling pathway may be the mechanism via which curcumin exerts its neuroprotective effects [84].

Inflammasomes are multiprotein complexes primarily responsible for regulating innate immunity. Among these, the NOD-like receptor pyrin domain-containing 3 (NLRP3) inflammasome is the most extensively studied, as its activation is associated with metabolic stress, inflammation, infections, and tissue damage. As a component of inflammasomes, NLRP3 detects damaged cell products and initiates immune responses. The NLRP3 inflammasome complex consists of three main components: a sensor protein (NLRP3), an apoptosis-associated speck-like protein containing a caspase recruitment domain (ASC), and the protease caspase-1.

One pathway for NLRP3 activation involves inflammatory bacterial products, such as lipopolysaccharides (LPS), which activate the NF-κB pathway. Curcumin has been shown to interfere with this pathway, suppressing NLRP3 inflammasome activation and the secretion of IL-1β, thereby modulating NF-κB signalling [85,86].

In addition to inflammasome-mediated effects, microglial pyroptosis—a form of pro-inflammatory programmed cell death driven by inflammasome activation—plays a critical role in neuroinflammation during stroke [87,88]. Notably, curcumin has been demonstrated to protect against stroke-induced neuronal damage by modulating microglial polarisation [87,89,90]. Recent in vivo and in vitro studies have shown that curcumin treatment reduces white matter damage caused by stroke while attenuating microglial pyroptosis. Curcumin inhibits NLRP3 activation induced by ischemic stroke in animal models of transient middle cerebral artery occlusion/reperfusion via upstream regulatory mechanisms. Specifically, curcumin suppresses NF-κB signalling, which prevents NF-κB-mediated NLRP3 activation and directly regulates microglial pyroptosis through this pathway. This ultimately results in reduced white matter damage caused by ischemic stroke [89].

Furthermore, curcumin influences additional aspects of the disease process, including modulation of microglial activity, antioxidant effects, and attenuation of neuroinflammation through NLRP3 inhibition [87].

Ischemia-reperfusion injury during stroke causes reactive oxygen species (ROS) to be produced and reactive nitrogen species (RNS), causing oxidative damage to lipids, proteins, and deoxyribonucleic acid (DNA). This oxidative stress aggravates neuronal injury and adds to the disruption of cellular homeostasis and the progression of stroke pathology with a robust inflammatory response characterised by the initiation of microglia, astrocytes, and infiltrating immune cells [91]. Pro-inflammatory cytokines, like tumour necrosis factor-α (TNF-α), IL-1β, and IL-6, are upregulated, contributing to neuronal damage, BBB disruption, and the recruitment of immune cells into the brain parenchyma [92,93]. Moreover, the ischemia activates apoptotic pathways, comprising the intrinsic (mitochondrial) and extrinsic (death receptor-mediated) pathways, resulting in the initiation of caspases and the cleavage of cellular substrates, ultimately resulting in neuronal apoptosis and tissue damage [94].

Being a strong antioxidant, curcumin scavenges free radicals, attenuates lipid peroxidation, and restores endogenous antioxidant defences, thereby counteracting oxidative stress-induced damage. Additionally, curcumin poses anti-inflammatory properties by inhibiting the activation of NF-κB and dropping the production of pro-inflammatory cytokines, like TNF-α and IL-1β, IL-6 [95]. By modulating microglial activation and suppressing the infiltration of peripheral immune cells, curcumin attenuates neuroinflammation and mitigates secondary brain injury following ischemic insult [96].

Moreover, curcumin exhibits neuroprotective properties through its ability to modulate excitotoxicity and apoptosis. By regulating glutamate receptors and calcium homeostasis, curcumin attenuates neuronal hyperexcitability and prevents excitotoxic cell death [97]. Furthermore, curcumin inhibits apoptotic ways by adjusting the expression of Bcl-2 family proteins, caspases, and apoptotic regulators, thereby promoting neuronal survival.

Curcumin has been observed to preserve BBB integrity by inhibiting matrix metalloproteinase 9 (MMP-9) [98] and reducing endothelial permeability, thereby limiting neuroinflammatory responses and reducing edema formation. Curcumin limited the extravasation of Evans blue, MMP-9 mRNA and protein expression, and the amount of Iba-1-positive microglia [99]. Further, curcumin cure increases the expression of the tight junction proteins, such as zonula occludens-1 and occluding, and helps improve ischemic stroke injury by keeping the integrity of the BBB [100]. Some scholars observed that long-term treatment with curcumin-induced neuroglobin expression might contribute to curcumin neuroprotective and neuroregenerative effects after brain ischemic events [101].

**Figure 2:**
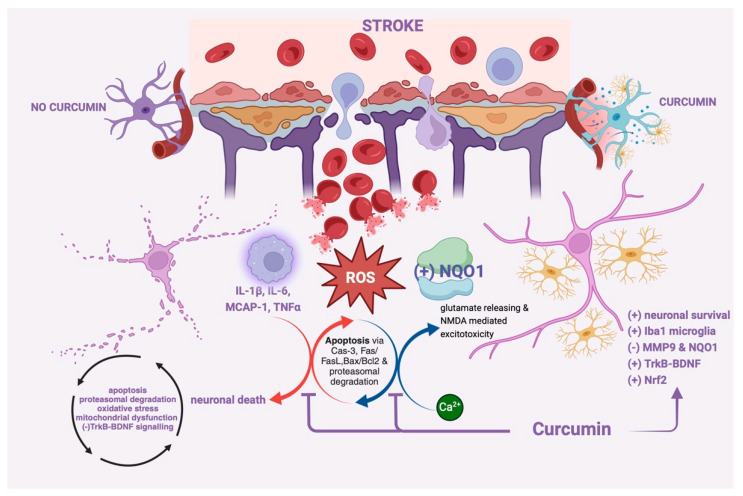
Curcumin effects in modulation of the pathophysiological reactions in haemorrhagic and haemorrhage-related stroke. The blood−brain barrier (BBB) is passed by erythrocytes and BBB structure is altered. Erythrocytes release the haemoglobin and BBB cells react by activating the inflammatory response via IL−1β, IL−6, MCAP−1 (monocyte chemoattractant protein−1), and TNF−α. The cytokine conspiracy determines free radical production, activation of the Cas-3 via Fas and Fas ligand (FasL), and Bax/Bcl2 signalling. Curcumin decreases expression of IL−1β, IL−6, MCAP−1, and TNF−α, and stimulates cell survival by diminishing or preventing the apoptosis processes. Curcumin inhibits NQO1, and MMP9 activities, hence promoting a tissue-protective and regenerative effect associated with increasing expression of the TrkB-mediated BDNF. IL-1β, interleukin 1β, IL−6, interleukin 6, MCAP−1, monocyte chemoattractant protein−1, TNF−α, tumour necrosis factor α, Cas−3, caspase 3, Fas and Fas ligand (FasL), a member of the TNF superfamily, a type II membrane protein, NQO1, NAD(P)H: quinone oxidoreductase 1, MMP9, matrix metalloproteinase 9, TrkB, tropomyosin-related kinase receptor type B, BDNF, brain-derived neurotrophic factor, ROS, reactive oxygen species.

Figure 2 summarises these curcumin effects in stroke and ischemic-brain issues. Strategies to enhance curcumin bioavailability in the CNS, such as nanoparticle formulations, liposomal delivery systems with brain-derived phospholipids, and co-administration with adjuvants like piperine, are being explored to surpass these limitations and develop the healing profile of the curcumin [102,103].

The clinical application of curcumin for neurological disorders continued to show promise but yielded mixed results. Clinical trials examined curcumin’s effects on conditions like Alzheimer’s, Parkinson’s, and migraines, revealing potential benefits in reducing oxidative stress and inflammation. The therapeutic benefits of curcumin in treating AD also include its protective effects on patients prone to strokes, that is, by inhibiting an increase in lipid peroxidation levels and nitric oxide synthesis [104]. While some studies demonstrated improvements in clinical outcomes such as reduced severity of migraines and motor function in Parkinson’s disease, the evidence remained inconsistent. Side effects were generally mild, with gastrointestinal issues reported in a few trials. Overall, curcumin’s therapeutic potential was acknowledged, though further research was needed to clarify its effectiveness. In the studies included in the systematic review, various interventions were assessed for their effects on patients with neurological disorders. For instance, in the study by Abdolahi in 2019 [105], 74 migraine patients were randomly assigned to receive either omega-3 fatty acids combined with nano-curcumin or a placebo for two months, resulting in a significant reduction in mRNA expression of COX-2 and iNOS, and a decrease in the frequency of attacks in the intervention group compared to placebo. In another study by Abdolahi in 2018 [106], a similar group of 74 migraine patients received omega-3 fatty acids and nano-curcumin, leading to a significant decrease in serum IL-6 levels. The 2021 study by Abdolahi [107] indicated that the frequency of headache attacks decreased significantly across all three intervention groups, which included combinations of omega-3 fatty acids and nano-curcumin. Ahmadi’s 2018 study on 54 patients with ALS revealed that nano-curcumin improved survival curves compared to placebo, although other outcomes did not show significant changes. In the 2008 study by Baum, 27 patients with Alzheimer’s disease receiving curcumin showed no significant differences in clinical outcomes compared to placebo. Chico’s 2018 study [108] on 42 ALS patients demonstrated that curcumin supplementation resulted in a stable ALS functional rating scale score, while the control group experienced a decline. The 2020 study by Djalali [109] reported that 38 migraine patients receiving nano-curcumin had significant decreases in PTX3 serum levels. In contrast, a study involving glioblastoma patients treated with micellar curcuminoids for four days showed a notable increase in intratumoural inorganic phosphate levels. The study by Ye in 2007 [110] involving 124 patients with Parkinson’s disease found that treatment with Zishenpingchan granules significantly reduced motor complications compared to the control group. Overall, the interventions demonstrated varying degrees of efficacy across different neurological conditions, with some studies reporting significant improvements in clinical outcomes and inflammatory markers, while others indicated no beneficial effects [105,106,107,108,109,110,111,112,113,114,115,116,117,118,119,120,121,122] cited by Mohseni et al. (2021) [123].

### 3.2. Curcumin Effects on Brain Microangiopathy

Microangiopathy refers to a group of disorders characterised by damage to small blood vessels, typically arterioles, capillaries, and venules. These conditions can lead to impaired blood flow, brain ischemia, and brain damage. Curcumin has demonstrated potential therapeutic effects in microangiopathy through various mechanisms [124].

Chronic inflammation contributes to endothelial dysfunction, vascular remodelling, and the progression of microvascular damage. Curcumin inhibits the activation of inflammatory pathways, such as NF-κB and cyclooxygenase-2 (COX-2), reduces the production of pro-inflammatory cytokines and chemokines, and improves ageing-related cerebrovascular dysfunction via the AMPK (AMP-activated protein kinase)/UCP2 (uncoupling protein-2) pathway.

The same authors observed that giving aged rats dietary curcumin for a month improved their defective cerebrovascular endothelium-dependent vasorelaxation. Curcumin decreased vascular oxidative stress, increased eNOS and AMPK phosphorylation, and upregulated UCP2 (mitochondrial uncoupling protein 2, which is essential for controlling ROS generation) in the cerebral arteries of aged rats and cultured endothelial cells [125].

By suppressing inflammation, curcumin may help preserve vascular integrity and function in microangiopathy [126]. Associated with inflammation, oxidative stress contributes significantly to the pathogenesis of microangiopathy, contributing to endothelial dysfunction, vascular injury, and impaired blood flow regulation. Curcumin scavenges free oxygen and functions as a strong antioxidant and nitrogen radicals, inhibiting lipid peroxidation, and upregulating endogenous antioxidant enzymes like GPx, CAT, SOD, Px (peroxidase), NADPH-reductase as well as maintaining cysteine and GSH cytoplasmic levels [127]. By reducing oxidative damage, curcumin helps protect blood vessels from injury and dysfunction, thereby preserving microvascular health by modulating endothelial cell metabolism.

**Figure 3:**
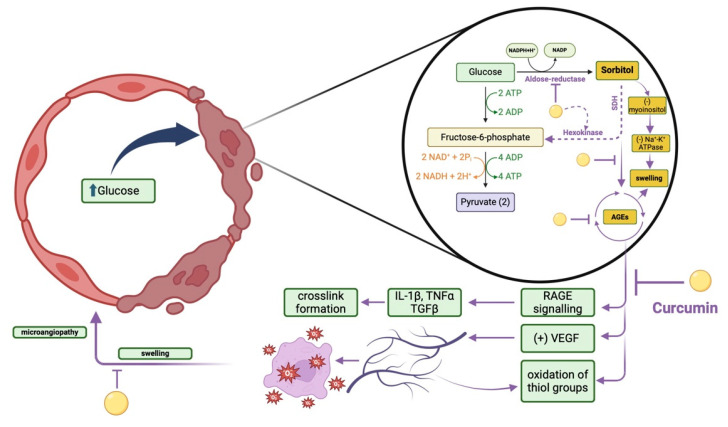
Curcumin prevents sorbitol-induced cerebral angiopathy associated with endothelial swelling. Regularly, high blood glucose determines the activation of the sorbitol synthesis with aldose-reductase catalysis. Sorbitol can be then converted to fructose-6-phosphate but, sorbitol accumulates in the cytoplasm and determines endothelial dysfunction, lowering the blood–brain barrier performance, decreasing myoinositol level, and increasing advanced glycation end (AGEs) production. These glycated molecules activate receptors for advanced glycation end products (RAGE) signalling and induce cytokine formation such as IL−1β, TNF−α, and TGF−β. AGEs also induced VEGF mRNA and protein expression, oxidation of the thiol groups, and oxidative damage of the vascular wall and near blood vessel brain tissue. Curcumin prevents sorbitol formation by inhibiting the aldose-reductase and then decreasing the AGEs concentration. By these actions, curcumin reduced VEGF signalling, decreased oxidative stress, protected the eNOS function, and maintained vascular homeostasis via endothelial cell protection. IL−1β, interleukin 1β, IL−6, interleukin 6, AGEs, advanced glycated endo-products, RAGE, receptor for AGE, TNF−α, tumour necrosis factor α, TGF−β, transforming growth factor−β, a pleiotropic cytokine involved in suppressive and inflammatory immune responses, VEGF, vascular endothelial growth factor, eNOS, endothelial nitric oxide synthase.

Dysfunction of the endothelium is a hallmark feature of microangiopathy. It has been demonstrated that curcumin protects endothelial cells from harm by enhancing nitric oxide (NO) production and promoting endothelial cell survival by inhibiting endothelial cell apoptosis [128]. By preserving endothelial function, curcumin helps maintain vascular homeostasis and prevent microvascular complications such as angiopathies. Curcumin has been shown to reduce vascular permeability by stabilising endothelial cell junctions, inhibiting the expression of vascular permeability/growth factors (VPF/VEGF), and suppressing inflammation-induced leakage [129,130]. By preserving vascular barrier function, curcumin helps prevent fluid extravasation and tissue damage associated with microangiopathy. In turn, the diabetes-related microangiopathy induced by sorbitol accumulation in endothelial cells via aldose-reductase action was observed as curcumin-prevented or pathology. The curcumin actions on endothelial homeostasis are synthesised in Figure 3.

Curcumin and curcumin analogues such as compounds with tetrahydroxy groups, demonstrated exceptionally strong aldose reductase inhibitory effects on aldose reductase and reducing effect on sorbitol accumulation [131]. Inhibition of phosphatidylinositol 3-kinase (PI3K) and p38 mitogen-activated protein kinase (MAPK) substantially reduced the *AR* gene’s promoter activity and curcumin-augmented mRNA levels. Luciferase reporter tests demonstrated that curcumin responsiveness required an osmotic response element in the promoter. Curcumin augmented the nuclear translocation of nuclear factor-erythroid 2-related factor 2 (Nrf2), and overexpression of Nrf2, but not the dominant negative Nrf2, enhanced the promoter activity of the *AR* gene.

By lowering reactive aldehydes, curcumin-induced increased AR activity may lessen cellular damage, but it may potentially hasten the onset of diabetes complications. The pleiotropic effect of curcumin under hyperglycemia needs to be additionally investigated [131].

For peripheral artery disease, curcumin enhances blood flow and reduces pain associated with intermittent claudication. Its anti-inflammatory properties contributed to improved vascular health and better oxygenation of tissues. A study performed in 2004 (Ramaswami et al.) showed that curcumin blocked the formation of homocysteine in endothelial cells of the coronary arteries [132]. Hyperhomocysteinaemia, characterised by elevated levels of homocysteine in the blood, was recognised as a significant vascular risk factor due to its role in promoting endothelial dysfunction, oxidative stress, blood clot formation, and atherosclerosis. Elevated homocysteine levels contributed to an increased risk of cardiovascular diseases, including coronary artery disease, stroke, and venous thromboembolism. It was found to be linked to genetic mutations, such as those in the *MTHFR* gene, and often associated with deficiencies in B vitamins like folic acid, B6, and B12. Management typically involved B-vitamin supplementation and dietary changes, although the direct causality between homocysteine levels and cardiovascular events remained debated, with some studies showing benefits and others not [133,134]. Posttransplant arteriopathy (PTA) was characterised by endothelial dysfunction, smooth muscle cell proliferation, and extracellular matrix remodelling. Endothelial damage occurred due to ischemia-reperfusion injury, inflammation, and immunosuppressive drugs, leading to increased pro-inflammatory cytokines. These cytokines activated endothelial cells, promoting leukocyte adhesion and smooth muscle cell migration. This resulted in intimal hyperplasia and narrowing of the arteries, impairing graft blood flow. Oxidative stress and renin-angiotensin system activation further contributed to vascular damage and fibrosis, ultimately compromising long-term graft survival. A comprehensive review published by Froldi and Ragazzi (2022) [135,136] observed curcumin as a main natural preventive molecule in AGEs-dependent endothelial dysfunction (diabetic arteriopathy) as well as in PTA. However, the mechanisms were relatively similar because, in both types of arteriopathies, curcumin was observed as an antioxidant, AGEs-formation inhibitor, and antithrombotic agent by decreasing the expression of adhesion molecules.

## 4. Curcumin in Neurodegeneration, Neuroinflammation and Schizophrenia

Neurodegenerative diseases are characterised by the gradual loss of neurons and cognitive or motor dysfunction, often associated with the build-up of misfolded proteins, oxidative stress, neuroinflammation, mitochondrial dysfunction, and impaired synaptic transmission (Table 1). Curcumin’s many-sided pharmacological characteristics make it a favourable candidate for neuroprotection and disease modification in neurodegenerative disorders.

### 4.1. Curcumin Effects in Alzheimer’s Disease

Curcumin exhibits diverse mechanisms of action in combating Alzheimer’s disease (AD), as detailed below.

#### 4.1.1. Inhibition of Aβ Formation

Curcumin has been shown to inhibit amyloid-beta (Aβ) production through several mechanisms. In mice models, intragastric administration of curcumin downregulated BACE1 expression, the enzyme responsible for cleaving amyloid precursor protein (AβPP) into Aβ. Similarly, in vitro studies confirmed that curcumin effectively inhibits BACE1 activity [10]. In a rat AD model, oral administration of curcumin reduced hippocampal Aβ accumulation and improved cognitive performance in tasks such as the passive avoidance test and the Morris water maze, a measure of spatial learning and memory. Additionally, curcumin reduced toxicity induced by various Aβ conformers, including monomeric, oligomeric, prefibrillar, and fibrillar forms. However, it is important to note that these findings were primarily derived from in vitro studies, which may not fully translate to in vivo scenarios [137].

#### 4.1.2. Copper Chelation

Copper contributes to neurological decline in AD, possibly by promoting Aβ plaque formation. Copper binds to Aβ peptides, creating an inter-strand histidine brace that facilitates the beta-sheet structure characteristic of plaques. It also catalyses the Fenton reaction, generating reactive oxygen species (ROS) such as hydroxyl radicals and superoxide anions, which exacerbate oxidative damage. Curcumin has demonstrated metal chelation properties, as evidenced by Picciano and Vaden’s study [138], which showed that curcumin chelates copper in the presence of Aβ peptides. However, its chelation efficiency appears to be concentration-dependent, performing optimally at lower concentrations].

#### 4.1.3. Cholesterol-Lowering Effects

Curcumin inhibits sterol regulatory element-binding proteins (SREBPs), transcription factors that regulate enzymes involved in glycolysis, lipogenesis, and cholesterol synthesis. SREBP-1 activation has been linked to neurotoxicity. In a study where rats were fed a high-fat diet supplemented with curcumin, the curcumin-treated group exhibited significantly lower serum triglycerides, total cholesterol, and LDL-cholesterol compared to controls. These effects were attributed to curcumin’s ability to upregulate cholesterol 7α-hydroxylase, the enzyme critical for converting cholesterol into bile acids. This mechanism also increased faecal triglyceride and cholesterol excretion [139].

#### 4.1.4. Anti-Inflammatory Activity

Curcumin modulates inflammation in AD by inhibiting M1 microglial activation, altering signalling pathways, and suppressing the secretion of pro-inflammatory molecules. Shi and colleagues [140] reported that curcumin blocked extracellular signal-regulated kinase 1/2 (ERK1/2) and p38 MAPK signalling in Aβ-activated microglia in vitro. This led to a significant reduction in the expression and production of pro-inflammatory cytokines, including TNF-α, IL-1β, and IL-6. Curcumin also enhanced microglial viability and mitigated Aβ-induced microglial activation.

Curcumin’s possible therapeutic benefits for AD disease have drawn a lot of interest. AD is a progressive neurodegenerative disorder characterised by the build-up of Aβ plaques, neurofibrillary tangles (NFTs) composed of hyperphosphorylated tau protein, neuroinflammation, oxidative stress, and synaptic dysfunction, leading to cognitive decline and memory impairment [141].

Here are some of the effects of curcumin in AD’s disease:

(i) Aβ clearance: Curcumin has been shown to modulate the aggregation and clearance of Aβ peptides, which are thought to be essential in AD pathogenesis. Curcumin has been described to hinder the formation of Aβ fibrils, destabilise preformed fibrils, and promote the clearance of Aβ aggregates by enhancing microglial phagocytosis and proteasomal degradation pathways [142,143,144].
(ii) Tau protein modification: Curcumin exhibits anti-tau properties by inhibiting the hyperphosphorylation of tau protein, a process linked with the formation of NFTs in AD brains [145]. Curcumin has been shown to inhibit kinases participating in tau phosphorylation, like glycogen synthase kinase-3β (GSK-3β), and promote tau dephosphorylation, thereby attenuating tau pathology and neuronal dysfunction [146].
(iii) Anti-inflammatory effects: Chronic neuroinflammation is a hallmark feature of AD, having a role in neuronal impairment and cognitive deterioration. Curcumin possesses powerful anti-inflammatory properties by hindering the activation of microglia, and the resident immune cells of the brain, and suppressing the fabrication of pro-inflammatory cytokines and chemokines, including IL-1β, TNF-α, and IL-6 [147,148,149].
(iv) Antioxidant Activity: Oxidative stress is essential in the pathogenesis of AD, contributing to neuronal destruction and the progression of neurodegeneration. Curcumin acts as an effective antioxidant by scavenging free radicals, inhibiting lipid peroxidation, and upregulating endogenous antioxidant enzymes, like superoxide dismutase (SOD) and catalase (CAT), thereby reducing oxidative damage and protecting neurons from oxidative stress [150,151].

The literature also highlights the limitations of curcumin applications in neurodegenerative diseases. In particular, the curcumin’s hydrophobicity raises the prospect of BBB penetration and buildup in the CNS. Curcumin’s poor water solubility and stability in solution are the primary causes of its incredibly low bioavailability, and quick intestinal first-pass and liver metabolism.

The amount of curcumin in the brain is still small, despite the numerous attempts being made to increase its bioavailability. Additionally, even though curcumin has moderately low toxicity when used at large doses—which is frequent in numerous in vivo and clinical studies—it may have some risks that have not been adequately discussed [152].

(v) Protection and synaptic function: Curcumin exerts neuroprotective effects by preserving neuronal viability, enhancing synaptic plasticity, and promoting neuronal survival. Curcumin has been shown to shield neurons against excitotoxicity, mitochondrial dysfunction, and apoptosis, while also promoting the expression of neurotrophic factors, like brain-derived neurotrophic factor (BDNF), which play a critical role in synaptic function and neuronal survival [153,154].
(vi) (BBB) Integrity: Disruption of the BBB contributes to neuroinflammation and neuronal damage in AD. Curcumin has been reported to preserve BBB integrity by reducing endothelial permeability, inhibiting matrix metalloproteinases (MMP2, MMP9) activity, and modulating tight junction proteins, thereby limiting the pass of peripheral immune cells and inflammatory mediators into the brain parenchyma [155,156,157].

Despite encouraging findings from preclinical research and a few clinical trials about curcumin’s possible therapeutic benefits in AD, more investigation is required to clarify the drug’s ideal dosage, bioavailability, and long-term safety profile, as these were identified as curcumin’s drawbacks [158].

Additionally, the development of curcumin formulations with improved bioavailability, such as nanoparticle-based delivery systems, may enhance its efficacy as a beneficial agent for AD [159].

Overall, curcumin represents a promising natural compound for the management of AD, targeting multiple pathological mechanisms involved in neurodegeneration and cognitive decline. Beyond its anti-inflammatory and antioxidant effects, curcumin could exert valuable effects on neurotransmitter systems dysregulated in schizophrenia, such as the dopaminergic, glutamatergic, and serotonergic pathways [160].

### 4.2. Curcumin Effects in Parkinson’s Disease

After AD, Parkinson’s disease (PD) is the second most common neurological illness. Pathological characteristics that set the disease apart include dopaminergic nigrostriatal neuronal degeneration, α-protein phosphorylation, proteinaceous inclusion development, Lewy bodies in neurons, and Lewy neurites in axons and dendrites.

Mechanistically, dopaminergic neuronal degeneration has been connected to elements like oxidative stress, protein misfolding due to genetic mutations, ROS from toxin exposure, and neuroinflammation (Table 1) [161,162,163,164]. Research on the population worldwide has shown that the usage of turmeric by Asian Indians, which contains the active ingredient curcumin, may contribute to the decrease in the occurrence of neurological illnesses in India compared to Caucasians [165].

Curcumin has been in use for decades as an antiseptic, anti-inflammatory, anti-bacterial, and anti-tumour agent against disorders like lung fibrosis, diabetes, wound healing, and arthritis, and for the reduction in blood cholesterol [166,167]. Dietary protocols with curcumin protect rats against cognitive deficits and synaptic dysfunction in some of them or in reducing neuronal apoptosis caused by stroke. Given that curcumin has been demonstrated to target several pathways, it could possess therapeutic benefits for PD.

In 6-OHDA (6-hydroxydopamine) rat models of PD, its neuroprotection may raise striatal dopamine levels, which have been demonstrated to be lower in PD cases [168,169]. Rajeswari and his colleagues as shown in Mythri and Bharath’s review have reported that in a mice model with MPTP (1-methyl-4-phenyl-1,2,3,6-tetrahydropyridine), curcumin helped increase the striatal dopamine and DOPAC levels (3,4-dihydroxyphenylacetic acid), a metabolite of the neurotransmitter dopamine [163,170]. It has been proposed that curcumin controls proteins related to iron metabolism.

One worth mentioning risk factor for the aetiology of PD is elevated brain iron concentration. There is more iron in the SN (*substantia nigra*) of PD brains and not only iron is accountable for the generation of OH radicals, but also responsible for auto-oxidation in the dopamine of the SN neurons causing the release of peroxide of hydrogen [171]. Therefore, curcumin reduces iron-induced toxicity without eliminating the iron intake. According to certain theories, curcumin binds Fe^2+^ and Fe^3+^ and prevents iron’s redox cycling and iron-induced lipid peroxidation, hence lowering oxidative stress [172]. Studies have suggested that curcumin regulates proteins involved in iron metabolism, a significant risk aspect in the progress of neurodegenerative diseases. By reducing iron-induced toxicity without eliminating iron intake, curcumin may help lower oxidative stress by binding to Fe^2+^ and Fe^3+^ ions, preventing iron’s redox cycling and lipid peroxidation. Research has also shown that curcumin can protect SH-SY5Y neuroblastoma cells from the cytotoxic effects of aggregated α-syn by reducing intracellular ROS, inhibiting caspase-3 activation, and decreasing apoptotic signals [173].

Oxidative stress contributes significantly to ageing and is related to several neurodegenerative illnesses, like PD. In PD, dopaminergic neurons aggregate and degenerate in response to an increase in ROS. Studies have shown that the extracellular addition of oligomeric α-syn to SH-SY5Y cells causes apoptosis, while curcumin, efficiently suppresses caspase-3 activation and mitigates these symptoms. Moreover, curcumin has been found to diminish ROS development and intracellular α-syn overexpression. Additionally, they showed that curcumin lessens the formation of ROS and intracellular overexpression of α-syn [174].

Chronic curcumin administration (200 or 400 mg/kg b.w.) also significantly improves memory and spatial learning because of the anti-inflammatory characteristics of curcumin on the brain. It also supplements redox state indicators like reduced GSH, CAT, SOD, and nitrite anions. Despite its generous beneficial effects on organisms, curcumin has some disadvantages already mentioned so it should be encapsulated in nanoparticle medication delivery systems for growth stability [175]. Encapsulation of curcumin in nanoparticle drug transport systems is recommended to enhance its stability.

**Figure 4:**
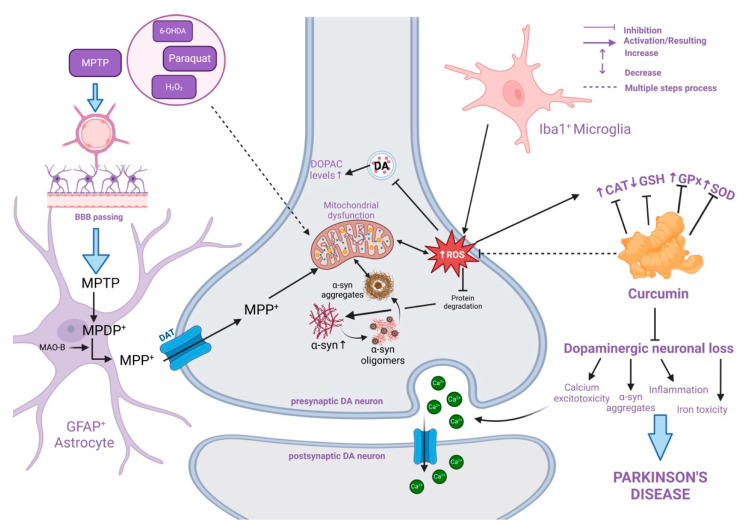
Phenotype is prompted by several factors (6-OHDA, MPTP, paraquat, H_2_O_2_) and the prophylactic role of curcumin in modulating the cellular process of PD. Even with the low bioavailability that was observed for curcumin, long-term treatment with curcumin or long-term exposure to turmeric developed a beneficial brain microenvironment, such as decreased calcium excitotoxicity, inflammation, and iron toxicity due to curcumin metabolites that pass more effectively the BBB than curcumin. Their action is characterised by α-syn disaggregation, β-amyloid clearance, maintaining the antiapoptotic feature of the brain microenvironment, and promoting neuronal survival as well as BBB regeneration by anti-inflammatory, antioxidant, and anti-angiopathic properties. MPTP 1-Me-4-Ph-1,2,3,6-tetrahydropyridine, MPDP^+^ 1- Me -4-Ph-2,3-dihydropyridine, MPP^+^ 1-Me-4-phenylpyridine, MAO-B monoamine oxidase B, GFAP+ glial fibrillary acidic protein, DAT dopamine transporter, DOPAC 3,4-dihydroxyphenylacetic acid, α-syn alpha-synuclein, ROS reactive oxygen species, DA dopamine, Iba1+ ionised calcium-binding adaptor molecule 1, CAT catalase, GSH glutathione, GPx glutathione peroxidase, SOD superoxide dismutase.

PINK1 (phosphatase and tensin homologue-induced kinase 1) is a significant risk factor for genetic alterations resulting in neuronal cell death seen in patients with PD. It plays a vital role in maintaining mitochondrial health. In a study by Van der Merwe (2017), a PINK1-deficient cellular model of PD was employed to scrutinise the influence of curcumin on mitochondrial activity. PINK1 siRNA was employed to cure SH-SY5Y neuroblastoma cells to reduce the expression of this protein. They examined the effects of curcumin with and without paraquat, an additional stressor that is known to cause oxidative stress-induced parkinsonism [172]. Reduced PINK1 expression in their knockdown model led to mitochondrial malfunction, which raised cell mortality and lowered cell viability—as would be predicted for the in vitro system employed in this investigation from the beginning. Figure 4 summarises the pathophysiological mechanism of PD and the protecting properties of curcumin as were observed in in vivo and clinical studies.

#### Experimental Models for PD

(I) Paraquat and Rotenone

Numerous detrimental effects of paraquat were demonstrated to be “rescued” by curcumin. It is suspected that curcumin may not be able to rescue paraquat-exposed cells with reduced PINK1 expression due to the severity of the damage [176]. Curcumin effects are heightened by synergistic combinations with other compounds such as piperine or resveratrol that have the same useful influence on ROS and RNS [177,178,179,180,181]. Curcumin is a dietary additive that has been extensively tested for toxicity and studied in preclinical settings using rats, mice, dogs, and primates due to its potential for cancer chemoprevention. Patients have not seen any negative side effects at dosages between 2000 and 8000 mg/kg b.w./day in clinical studies. Curcumin treatment decreased the α-syn accumulation in A53T cells and improved the aggregate cytoplasmic clearance. Curcumin might cure macro autophagy and cut the level of α-syn in A53T cells.

All these results once more indicated that curcumin is reasonably safe, has no adverse effects when the recommended dosage is respected, and is an encouraging healing candidate in neurodegenerative diseases [176].

Curcumin, interestingly, caused a notable rise in total cellular glutathione levels, which enhanced defence against nitrosative stress. Glutamate-cysteine ligase is the rate-limiting enzyme in glutathione production, and curcumin induces its expression, which raises glutathione levels. When extracted from N27 cells and cultured with 0.5 µM curcumin, mitochondria demonstrated defence against peroxynitrite. Mice given intraperitoneal doses of curcumin (50 mg/kg b.w. for 1-3-5 days) showed a substantial intensification in brain levels of total glutathione. Mice given a curcumin injection also showed protection from peroxynitrite poisoning in their whole brain mitochondria.

These findings imply that curcumin directly detoxifies to protect against PN in vitro, while indirectly protecting against PN in vivo by raising glutathione levels in cells, which detoxify PN [182]. In their work, Abrahams and colleagues highlighted the fact that the cause of most PD instances is unidentified, even though genetic mutations in genes related to other processes, such as mitochondrial health (PRKN, PINK1, LRRK2, and DJ1), account for about 5–10% of instances; after compiling 64 studies, they computed that 11 of them examined the therapeutic potential of curcumin against oxidative stress using cellular models of PD that had been transfected or treated with toxins [179,183].

In two studies curcumin derivatives were given to neuroblastoma cells, SH-SY5Y and SK-N-SH, before the cells were exposed to rotenone, a reputable model for PD. The authors of both researchers concluded that the curcumin derivatives suppressed oxidative stress generated by rotenone, which in turn decreased apoptosis.

Another study mimicked the neuronal damage seen in NDDs (neurodevelopmental diseases) by pre-treating SH-SY5Y cells with curcumin and then exposing them to paraquat [180,183,184].

Curcumin enhanced the expression of the antioxidant genes (SOD, GPx) and decreased ROS levels in cells exposed to paraquat [185]. Two more experiments included transfecting mutant α-syn into SH-SY5Y and PC12 cells and then incubating the cells with curcumin after the transfection. Curcumin was observed to reduce ROS generation mediated by α-syn in both trials. It should be highlighted, nevertheless, that curcumin had no impact on ROS when mutant α-syn was not produced in the cells. This might suggest that curcumin’s protective qualities only materialise in reaction to an oxidative stressor [186,187].

In studying the PD mechanisms, six researchers used animal models to examine curcumin’s potential. Out of these, two employed rotenone [188,189,190,191] to induce a PD phenotype, while the remaining ones used 6-OHDA, MPTP, paraquat, or H_2_O_2_ [192,193].

(II) 6-OH-Dopamine

Rats were given an oral pre-treatment of curcumin and its derivatives before receiving a neurotoxic 6-OHDA striatal injection. The 6-OHDA-induced decrease in the CAT, GSH, GPx, GR, and SOD was counteracted by curcumin and curcuminoids. Additionally, as indicated by lower MDA (malondialdehyde) levels, curcumin and its compounds decreased lipid peroxidation.

Consequently, in the 6-OHDA-treated rodents, curcumin decreased oxidative stress, a result linked to enhanced motor performance and prevention of neuronal loss. Notably, antioxidant activity was not increased to the level observed before treatment with curcumin or its derivatives. Thus, it would be intriguing to ascertain the length of time that curcumin provides protection [194]. Curcumin has been shown in other assessments from across the world to avoid the death of dopaminergic neurons. As a result, long-term use of curcumin may help reduce the deadly consequences of environmental chemicals that cause symptoms similar to PD by downregulating the signalling of genes or mTOR/p70S6K [195,196,197].

The production of neuromelanin from degenerating neurons prompts the activation of microglia, which consequently leads to the death of neurons. This is one of the several interrelated pathways contributing to the pathophysiology of PD [198]. This concurrently initiates the self-activating cycle of neurodegeneration by stimulating the synthesis of neuromelanin. It has been shown that even in the absence of further toxin exposure, an acute MPTP subjection can result in a long-lasting neurodegenerative consequence from MPTP-induced parkinsonism.

It has been demonstrated that polyphenols may modify cellular signalling pathways, including changes in the IRF (interferon regulatory factors), Nrf2, and Akt signalling pathways that connect DNA binding receptors to the NF-κB transcription factor [199]. By inhibiting phosphorylation or ubiquitination processes, polyphenols prevent the degradation of IkB, thus inhibiting the translocation of NF-κB into the nucleus and ultimately blocking the production of pro-inflammatory cytokines.

Moreover, polyphenols inhibit the binding of NF-κB subunits with target DNA, which further reduces inflammation. By hindering the expression of several NF-κB regulated proinflammatory proteins (cytokines, chemokines) and enzymes (iNOS, COX-2), both mechanisms of action provide indirect protection. Dietary polyphenols, which include curcumin, are a type of secondary metabolites that regulate the expression of many proinflammatory genes [200,201,202].

The most prevalent glial cells in the CNS, astrocytes, play a significant role in both neurodegenerative illnesses. In 1-methyl-4-phenyl pyridinium ion (MPP^+^) encouraged mesencephalic astrocytes, curcumin demonstrated anti-inflammatory and antioxidant effects. Because curcumin inhibited CYP2E1 activity and reduced ROS, it showed cytoprotective effects in primary mouse mesencephalic astrocytes against toxicity caused by MPP^+^ and LPS [203].

In another extensive study about curcumin and 6-OHDA-induced PD, the authors observed that curcumin meaningfully decreased 6-OHDA-induced loss of TH-IR (tyrosine hydroxylase immunoreactive neurons) in the SNpc and TH-IR fibers in the *striatum* [204]. In the ipsilateral SNpc (*substantia nigra pars compacta*), an intrastriatal 6-OHDA lesion caused a 1/3 decrease in TH-IR neurons. In contrast, 6-OHDA animals treated with curcumin showed a mere 12% loss of nigral TH-IR neurons. Given that DMSO has been demonstrated to be neuroprotective in brain damage, the researchers investigated whether the neuroprotective effects of 6-OHDA/curcumin were due to the DMSO vehicle. It was discovered that 6-OHDA neurotoxicity was not altered by DMSO. In the SNpc area, 6-OHDA caused a 78% reduction in TH (tyrosine hydroxylase) immunoreactivity in the ipsilesional striatum. Curcumin dramatically decreased the 6-OHDA-induced rise in nigral glia (32% and 27% increase in Iba1-IR microglia and GFAP-IR astrocytes, respectively) in comparison to sham rats [205] TH-IR neuronal loss brought on by 6-OHDA or MPTP is reduced when microglial response is blocked.

Curcumin may, however, also exert its neuroprotective effects by inhibiting the glial response. Early glial response after brain damage may have a role in both protective and reparative processes but given the existence of site-specific glial activations in *Homo Sapiens* neurodegenerative illnesses like PD, it is plausible that this response also plays a decisive role in neuronal injury.

Curcumin’s ability to decrease acute and chronic nigrostriatal inflammation may be linked to its anti-inflammatory properties in PD. Curcumin’s wide availability, low toxicity, and range of pharmacological activities made it a prime candidate for several studies.

Curcumin’s restricted therapeutic window may be attributed to its low serum half-life of less than 30 min and extremely poor oral bioavailability [206]. Nonetheless, several papers have noted improved curcumin absorption after intraperitoneal administration.

(III) MPTP

In a mouse model of PD, curcumin administration over an extended period (2–5 months) reduced the neurotoxicity of MPTP by upregulating the levels of TGFβ1 (transforming growth factor beta 1) and GDNF (glial cell line-derived neurotrophic factor) [207,208]. According to Eghbaliferiz and colleagues’ published opinion, in line with Sian et. al., curcumin prevented glial cell activation, NO production, NF-B activation, and microglial NADPH oxidase (PHOX) activity in addition to reducing neuroinflammation in the LPS-induced PD model [197,209].

Curcumin protects the GSH synthesis, inhibits α-syn aggregation, suppresses the glial response, and prevents oxidative stress from exercising its neuroprotective action [210]. Furthermore, curcumin had a neuroprotective influence in a 6-OHDA hemi-Parkinson-mouse model by reducing Iba1-IR in microglia and GFAP-IR in astrocytes in the ipsilateral striatum and protecting nigrostriatal dopaminergic neurons [211].

Curcumin enhanced GPx and SOD activities and decreased oxidative stress caused by copper intake via the Wnt/β-catenin signalling pathway in the rodent model of PD. High copper intake was observed as a major factor in the pathophysiology of PD [212,213].

After MPP^+^ assaults, there is a report of overexpression of inflammatory cytokines. Yu et al. investigated how curcumin affected the synthesis of inflammatory cytokines in MPP^+^-stimulated astrocytes. Curcumin pretreatment decreased TNF- α and IL-6 secretion, but it also raised IL-10 levels in MPP^+^-exposed astrocytes [212,213].

(IV) Lipopolysaccharide

Lipopolysaccharide (LPS, 5 µg/5 µL PBS) injections into the SN of rats were used to create an animal model of PD.

Intraperitoneally curcumin (40 mg/kg b.w.) was then injected once a day for 21 days. The suppression of GFAP^+^ astrocytes activation by immunofluorescence and the activation of the NADPH oxidase complex by RT-PCR demonstrated the modulatory properties of curcumin. The same authors observed that inducible nitric oxide synthase, proinflammatory cytokines (TNF-α, IL-1β, and IL-1α), transcription factor NF-κB, and regulating molecules of the intrinsic apoptotic pathway (Bax, Bcl-2, caspase 3 and caspase 9) were all shown to be upregulated by curcumin in response to LPS stimulation [214].

**Figure 5:**
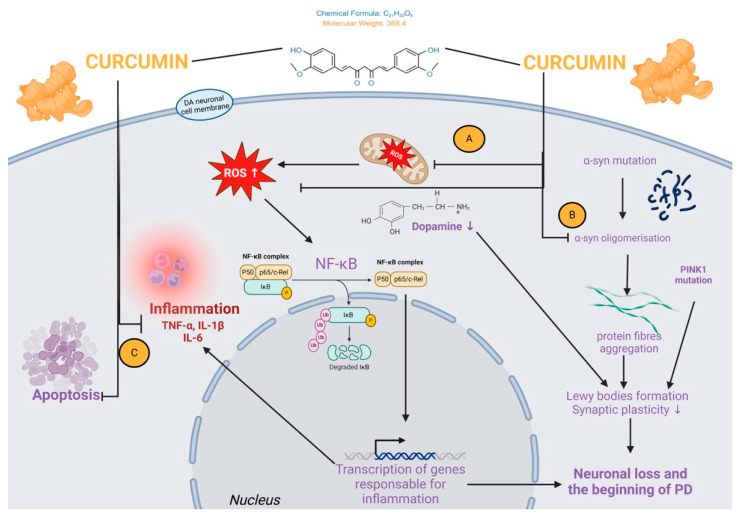
Curcumin interaction with α-syn aggregation and inflammatory response. A. Mitochondrial dysfunction generates ROS which stimulates gene transcription for inflammatory mediators via NF-κB. The presence of curcumin reduces the process of ROS scavenging. B. When mitochondria are lesioned, protein aggregation is stimulated by a lower pH value of the cytoplasm, ROS-mediated partial denaturation, and α-syn as a Parkinson’s disease inductor follows these rules. Also, curcumin decreases α-syn aggregation as a mitochondrial protective action. C. Definitely, curcumin reduces inflammation. The mechanisms probably include epigenetic control of TNFα, IL-1β, and IL-6 expression and signalling; however, more research is still required to clarify these mechanisms. DA dopamine, ROS reactive oxygen species, α-syn alpha-synuclein, PINK1 phosphatase and tensin homolog induced kinase 1, NF-ᴋB nuclear factor kappa-light-chain-enhancer of activated B cells, P50 p65/c-Rel family of NF-ᴋB dimers which generate inactive complexes that are sequestered in the cytosol, Iᴋβ inhibitor of NF-ᴋB, TNF-α tumour necrosis factor-alpha, IL-1β interleukin 1β, IL-6 interleukin 6.

Curcumin interactions with inflammation and apoptosis signalling pathways connected with α-syn aggregation and neuronal survival are described in Figure 5.

Curcumin’s multifaceted actions make it a promising therapeutic candidate. However, its limited bioavailability underscores the need for advanced formulations, such as nanoparticles or liposomal curcumin, to enable effective clinical application. Such effective clinical applications are stated in Table 1.

Curcumin has been extensively studied for its therapeutic benefits, including its well-documented anti-inflammatory and antioxidant properties. However, concerns about its potential hepatotoxicity have emerged, particularly in the context of dietary supplements and formulations designed to enhance its bioavailability. Case reports have identified turmeric-associated drug-induced liver injury (DILI), often characterised by elevated liver enzymes and hepatocellular damage. For instance, a study reported that seven acute hepatic events recorded in Tuscany, Italy, in 2019 were linked to *Curcuma longa*-induced hepatotoxicity. These incidents frequently involved high doses or formulations enhanced with piperine, a compound that significantly boosts curcumin absorption. The reported daily doses of *Curcuma longa* ranged from 250 to 1812.5 mg, with exposure periods spanning 2 weeks to 8 months [215].

Although curcumin is generally considered safe at recommended doses (up to 3 mg/kg/day, according to EFSA guidelines), there is evidence of idiosyncratic or dose-dependent liver injuries associated with prolonged or excessive use. The Roussel Uclaf Causality Assessment Method (RUCAM) has been employed in several cases to assess curcumin-related DILI, with some cases showing probable causation. However, factors such as the role of piperine, supplement quality, and individual variability complicate the assessment of curcumin’s direct hepatotoxic effects. Further research is needed to elucidate the mechanisms and risk factors underlying curcumin-associated hepatotoxicity [216,217].

### 4.3. Curcumin and Schizophrenia

Dopamine dysregulation is a hallmark feature of schizophrenia, with hyperactivity of dopaminergic transmission involved in the symptoms of the disorder, such as hallucinations and delusions. Preclinical studies have shown that curcumin modulates dopaminergic neurotransmission by inhibiting the activity of dopamine-degrading enzymes, like monoamine oxidase (MAO) and catechol-O-methyltransferase, and regulating dopamine receptor expression and signalling [218].

In addition to its effects on neurotransmitter systems, curcumin exhibits neurotrophic properties that could promote neuronal survival, synaptic plasticity, and neurogenesis, processes that are impaired in schizophrenia. Curcumin has been shown to upregulate the expression of BDNF, a key mediator of neuronal growth and survival, and stimulate adult hippocampal neurogenesis, which could support the development of cognitive abilities and mood stabilisation in schizophrenia [219,220]. As in AD, preclinical studies have delivered compelling confirmation for the beneficial perspective of curcumin in schizophrenia, clinical research in this area is still limited, and further investigation is needed to elucidate its efficacy, safety, and optimal dosing regimens in human subjects.

Dysregulated signalling of NMDA glutamate has been involved in the negative, positive, and cognitive symptoms of schizophrenia. Previous research suggests that curcumin exhibits neuroprotective effects and modulates neuroinflammation by targeting the NMDA system [221]. Curcumin has been shown to reverse structural alterations in the dendritic morphology of CA3 pyramidal neurons in the hippocampus in rodent models subjected to restraint stress. Additionally, it protects corticosterone-induced overexpression of NMDA receptor subunit NR-2B in hippocampal neurons [222].

Curcumin also mitigates glutamate accumulation, reduces NMDA receptor density, and downregulates the expression of the glutamate/aspartate transporter in the cerebral cortex. Furthermore, preclinical and clinical studies indicated its beneficial effects in addressing metabolic syndrome [223].

Challenges like curcumin’s reduced bioavailability and rapid metabolism necessitate the development of innovative formulations or delivery systems (e.g., nanoparticle formulations, liposomal delivery systems, and co-administration with adjuvants or brain-derived phospholipids) to enhance its absorption and retention in the brain. Additionally, well-designed clinical trials are warranted to evaluate the long-term effects of curcumin supplementation on symptom severity, cognitive function, general well-being, and functional outcomes in individuals with schizophrenia.

## 5. Curcumin Effects on Inflammation Caused by Diabetic Neuropathy

Diabetic neuropathy is a common and debilitating complication of diabetes mellitus, affecting both type 1 and type 2 diabetes patients. It is defined as nerve injury brought on by extended exposure to elevated blood sugar levels [224]. Peripheral, autonomic, proximal, and focal neuropathy are some of the different ways that diabetic neuropathy can present itself. The pathogenesis of diabetic neuropathy is complex and involves multiple mechanisms, including oxidative stress, inflammation, mitochondrial dysfunction, impaired nerve blood flow, and the development of progressive glycation end-products (AGEs) under aldose-reductase catalytic action. Moreover, the pathophysiology and progression of diabetic neuropathy may be explained by the following pathways: hexosamine, AGEs, PKC, PARP and MAPK, NF-κB, hedgehog, TNF-α, COX, interleukins, lipoxygenase, nerve growth factor, Wnt, autophagy, and GSK3 signalling [225].

The pathophysiology of diabetic neuropathy is significantly influenced by chronic inflammation, contributing to nerve damage and dysfunction. Curcumin holds strong anti-inflammatory properties in diabetic neuropathy status, inhibiting the activation of inflammatory pathways like NF-κB and reducing the production of pro-inflammatory cytokines and chemokines. By suppressing inflammation, curcumin may alleviate nerve inflammation and attenuate neuropathic pain in diabetic neuropathy [226].

Oxidative stress represents a central factor in nerve damage in diabetic neuropathy, arising from a disparity between the ROS and antioxidant defences via GSH/GSSG ratio and oxidoreductase activity production. As stated before, as a powerful antioxidant, curcumin scavenges free radicals, prevents lipid peroxidation, and increases endogenous antioxidant enzymes. By reducing oxidative damage, curcumin helps protect nerves from oxidative stress and preserves nerve function in diabetic neuropathy [227,228].

Curcumin also exerts neuroprotective effects by promoting nerve regeneration, enhancing nerve growth factor (NGF) expression, and protecting against neuronal apoptosis. Curcumin has been shown to stimulate neurite outgrowth, upsurge the expression of neurotrophic factors, and inhibit apoptotic pathways in nerve cells. By promoting nerve repair and regeneration, curcumin may help improve nerve function and alleviate symptoms of diabetic neuropathy [229]. Further, curcumin has been shown to modulate pain pathways by inhibiting nociceptive signalling, reducing the expression of pain-related neurotransmitters, and attenuating central sensitisation [230,231].

In diabetic neuropathy, the microvascular dysfunction contributes to nerve ischemia and damage.

Curcumin improves microvascular function by enhancing endothelial function via VEGF signalling, increasing nitric oxide (NO) bioavailability by eNOS protection, and reducing endothelial dysfunction [232]. At a molecular level, besides radical scavenging and anti-inflammatory effects in diabetic neuropathy, curcumin enhanced the levels of the defensive reactions based on Nrf2 signalling, peroxisome proliferator-activated receptor-γ (PPAR-γ), CCAAT/enhancer binding protein (C/EBP) homologous protein (CHOP), and activating transcription factor 3. Additionally, curcumin has been demonstrated to downregulate several transcription factors, comprising NF-κB, protein kinases, chemokines, and inflammatory biomarkers in diabetic neuropathy whereas curcumin effects in cancer were in the opposite direction [233,234].

These polarised aspects of curcumin’s effects on health and disease need more investigation taking into account the pharmacokinetic profile of the curcumin and its metabolism in the brain and other tissues as well as in tumours and stem cells. 

## 6. Curcumin Effects in Metal-Induced Neurotoxicity

Metal-induced neurotoxicity is a complex and multifaceted phenomenon that has garnered significant attention in both scientific and public spheres. Exposure to certain metals, whether through environmental pollution, occupational hazards, or dietary intake, has been linked to various neurological disorders and cognitive impairments [235].

Heavy metals like lead, mercury, and cadmium have been demonstrated to disrupt cellular redox balance and impair antioxidant defence mechanisms, exacerbating oxidative damage [236]. Additionally, metals can induce neuroinflammation by activating astrocytes, ependymal cells, and oligodendrocytes and encouraging the production of cytokines that promote inflammation and chemokines. This inflammatory response contributes to neuronal injury and synaptic dysfunction, further exacerbating neurotoxic effects (Table 1) [237,238].

Furthermore, certain metals possess the ability to disrupt neurotransmitter systems, interfere with synaptic transmission, and alter neuronal signalling pathways, thereby impairing cognitive function and behaviour [239,240]. Efforts to mitigate metal-induced neurotoxicity encompass various approaches, including environmental regulation, occupational safety measures, and public health interventions.

At the individual level, dietary modifications and nutritional interventions might aid mitigate the adverse effects of certain metals. For instance, consuming a diet containing high amounts of antioxidants and micronutrients can enhance cellular defence mechanisms against oxidative stress and mitigate neuroinflammation [239].

Chelation therapy, although controversial and primarily reserved for acute poisoning cases, may be considered in several circumstances to reduce the metal burden on the organisms.

Studies have shown that curcumin is rapidly metabolised both in cell culture conditions and in vivo, primarily through reduction and conjugation processes. Upon metabolism, curcumin undergoes consecutive reduction in the double bonds in the heptadienedione chain, resulting in the formation of di-, tetra-, hexa-, and octa hydro curcumin metabolites [13]. Among these reduced metabolites, tetra- and hexahydrocurcumin constitute the largest portion of the detected curcumin metabolites. These metabolites contribute to the diverse biological properties of curcumin, making the study of these metabolites crucial for understanding its full therapeutic potential [241].

It has been demonstrated that curcumin has protective properties for GSH, an antioxidant molecule, and a clearance enhancer for cell deposits of heavy metals [242,243].

The primary toxicity route involves the reaction between thiols and heavy metals, leading to GSH inactivation and necrosis [244]. Curcumin and curcuminoids have demonstrated the ability to protect GSH levels, and some studies have shown that curcumin can function as a metal scavenger [245], exerting neuroprotection and enhancing brain clearance processes of heavy metals by helping the elimination of metal ions via urine and faeces [246,247]. Hence, curcumin reduces the systemic burden of heavy metals and prevents their accumulation in vital organs, including the brain.

Thus, the protective effect of curcumin is attributed to its ability to neutralise free radicals and chelate metal ions [248]. Curcumin functions simultaneously as a metal chelator and antioxidant. Its unique chemical structure enables the formation of highly stable chelate complexes, effectively removing toxic metal ions and preventing the polymerisation of Aβ and the subsequent formation of harmful conformations. This chelation ability primarily stems from curcumin’s natural complexing properties and the presence of a 1,3-diketone group, which facilitates tautomeric transitions. The more stable enol form of curcumin acts as an effective complexing agent, binding strongly to metal ions and forming stable complexes. This makes it highly suitable for chelating ingested toxic metals. The keto-enol region serves as the reaction centre, protecting against reactive free radicals. The enol form’s stability is enhanced by charge delocalisation, allowing it to generate 1:2 and 1:3 chelates with metal ions. Due to its pronounced lipophilicity, curcumin can easily cross the blood–brain barrier and cell membranes, enabling it to neutralise toxic metals within cells [249]. On the other hand, its unique chemical structure allows it to form stable complexes with toxic metal ions, such as Al (III), copper, arsenic, cadmium, iron, lead, mercury, zinc, and selenium, effectively neutralising their harmful effects. Curcumin’s enol form plays a key role in metal chelation, protecting against oxidative stress and preventing the formation of harmful Aβ plaques associated with neurodegenerative diseases like Alzheimer’s [250]. In animal studies, curcumin has shown neuroprotective effects, improving memory, reducing oxidative damage, and lowering inflammation, particularly in conditions involving metal toxicity. It mitigates damage from metals like aluminium, arsenic, cadmium, copper, and lead, by facilitating their removal, reducing tissue accumulation, and enhancing antioxidant activity. Curcumin also protects against liver, kidney, and brain damage caused by these metals, including by inhibiting oxidative stress, modulating autophagy and apoptosis, and stimulating immune responses. In cases of mercury and selenium toxicity, curcumin reduces oxidative stress and improves biochemical balance, although excessive curcumin supplementation may interfere with essential mineral absorption. Its ability to cross the blood–brain barrier and reduce neurotoxic effects further highlights its potential for treating metal-induced neurodegenerative diseases. Overall, curcumin’s ability to scavenge free radicals, chelate metal ions, and stimulate antioxidant enzymes makes it a promising candidate for protecting against metal toxicity [245].

The IC_50_ value of curcumin in neurons and glial cells is a decisive parameter in pharmacology that represents the concentration of curcumin necessary to inhibit half of a specific biological activity.

Several studies noted different IC_50_ values of curcumin using normal and neoplastic brain-derived cell lines. When curcumin was complexed with manganese, the system exhibited pronounced capability to defend brain lipids peroxidation with IC_50_ range between 6.3 and 26.3 µM [251]. A study in which rodents were exposed to Cu^2+^ at 100 mg/kg b.w. revealed that curcumin as a neuroprotective agent has shown IC_50_ for this anti-peroxidative activity compound at the 66.3 nM value [252].

Studying the antiproliferative effect of curcumin in a medulloblastoma cell line (DAOY), curcumin decreased histone deacetylase 4 expression, amplified tubulin acetylation, and induced a G2/M cell cycle arrest with an IC_50_ value of 35 µM, after 48 h from exposure. In U87 medulloblastoma cell lines IC_50_ value for curcumin was 37.3 µg/mL as compared to chemotherapeutics such as etoposide (IC_50_ = 6.5 µg/mL) and temozolomide (IC_50_ > 2000 µg/mL) and Turmeric ForceTM (NewChapter, Brattleboro, VT, USA) with IC_50_ value measured at 30.8 µg/mL.

The cell lines U138MG, U87, U373, and C6 were exposed to curcumin, revealing IC_50_ values of 29, 19, 21, and 25 μM, respectively. In contrast, astrocytes exhibited an IC_50_ of 135 μM [253] (Table 2).

## 7. Current Challenges and Prospects Concerning Curcumin-Based Therapies

Future prospects may study the role of curcumin in the Warburg effects conditions as in the instance of cancerous cells as well as the curcumin actions on glutathione metabolism which acts as a tuner in curcumin metabolic effects. Further, intelligent curcumin formulations such as curcumin complexes with brain-derived phospholipids or curcumin complexes with various proteins or liposomal encapsulation could be a strategy to improve curcumin bioavailability, stability, and BBB passage with more confident effects in cerebrovascular diseases.

As a general role of dietary supplements, including natural compounds like curcuminoids such as curcumin, flavonoids, milk proteins, carbohydrates, fatty acids, amino acids, and minerals is intended to provide nutrients that may otherwise not be available in adequate quantities for healthcare and for therapeutic effect. To the best of our knowledge, our preliminary results [254,255,256,257] are the first reported on nanoencapsulation of curcumin within whey protein concentrate, when protein undergoes a process of self-assembly where the hydrophilic surface of the protein is outside into the aqueous environment, while the hydrophobic core is trapped inside the protein.

In consequence, this approach might provide an encouraging platform for improving the management strategy of neurodegenerative disorders, by using nanoparticle-mediated curcumin delivery to the brain, which requires exploration in vitro and in vivo in the foreseen prospects for prevention of neurodegenerative disorders and the human health benefits. Moreover, these future thorough investigations are required to assess the bio-efficacy of nanoparticles comprising curcumin, to extend these multi-functional nanoparticles for therapeutic applications and for functional food requested for healthcare, to enhance nutritional profiles while improving texture, taste, and shelf life.

Although the body of evidence regarding curcumin bioactivities is very exhaustive, curcumin is not a gold standard in plant-based therapies, but its observed effects in preclinical and clinical studies propose that it is still a natural compound that is not fully understood, and also that it is a prophylactic instrument in many cerebral disorders.

## 8. Conclusions

Curcumin’s effects on cerebrovascular homeostasis are not fully understood. There are two main perspectives regarding curcumin’s actions: its bioavailability and pleiotropic effects as mentioned in this review (downregulates pro-apoptotic factors like caspase-3, inhibits pro-inflammatory cytokines (such as TNF-α, IL-1β) and NF-κB signalling, increases brain-derived neurotrophic factor (BDNF) expression, reducing heavy metals-induced neurotoxicity, decreasing α-syn aggregation, increasing NO bioavailability by eNOS protection) correlated to the pharmacokinetic profile of curcumin based on a blood-to-brain absorption coefficient. It is suggested that the cerebrovascular actions of curcumin stem from high doses lead to the presence of its principal metabolites that are capable of crossing the blood–brain barrier, but smaller doses of curcumin can also be delivered through nanomedicine, utilising advanced formulations such as nanoparticles, liposomes, and polymer-based carriers to enhance its stability, bioavailability, and targeted delivery to the brain.

In the context of stroke, curcumin appears to alleviate inflammation and oxidative and nitrosative stress. It reduces ischemia and contributes to an optimal adaptive immune response through Iba1 microglia activation, decreasing matrix metalloproteinase 9, and enhancing Nrf2 expression. Curcumin’s vascular effects on endothelial cells, facilitated by its adherence and the presence of metabolites, enhance sorbitol clearance.

Curcumin also exhibits a vascular protective effect in type 1 and 2 diabetes, conditions linked with a bigger risk of neurodegeneration due to hyperglycemia’s inhibitory effect on proteasomal degradation. These actions suggest a probable part for curcumin in the complementary management of AD, PD, and microangiopathy, regardless of blood glucose concentrations.

Furthermore, curcumin exposure enhances VEGF expression while lowering RAGE signalling and oxidative stress in the vascular microenvironment. However, conflicting results regarding curcumin’s effects on normal and cancerous cells indicate a gap in understanding curcumin’s mechanisms and biological effect profiles.

According to Karlstetter et al. (2011), curcumin exerted significant anti-inflammatory effects on microglial cells by modulating their transcriptomic activity. It downregulated pro-inflammatory genes such as toll-like receptor 2, nitric oxide synthase 2, and prostaglandin-endoperoxide synthase 2, especially in LPS-stimulated BV-2 cells. Furthermore, curcumin has induced anti-inflammatory genes like peroxisome proliferator-activated receptor alpha (PPARα) and IL-4, promoting neuroprotection. These molecular changes reduced BV-2 microglial motility and migration, key features of neuroinflammation, and decreased their neurotoxic effects, suggesting again a therapeutic potential for neurodegenerative diseases [258]. Ullah et al. (2022) described how curcumin mitigated ageing-related changes in microglial cells. With ageing, microglia became primed, exhibiting heightened sensitivity to inflammatory stimuli and an exaggerated pro-inflammatory response. Curcumin actioned on the same canonic pathways (NF-κB and TLR4, reducing the expression of inflammatory mediators such as TNF-α and IL-1β) for reversing the morphological de-ramification observed in ageing microglia, restoring their ability to maintain central nervous system homeostasis and reducing chronic neuroinflammation [259]. Bernardo et al. (2021) observed that curcumin promoted the differentiation of oligodendrocyte progenitor cells (OPs) into mature oligodendrocytes (OLs) by activating the nuclear receptor PPAR-γ, which was demonstrated by the nuclear translocation of PPAR-γ and subsequent upregulation of mitochondrial proteins like PGC1-α and COX1. This activation facilitated the phosphorylation of ERK1/2, a kinase essential for OL differentiation, with partial dependence on PPAR-γ. Additionally, as was observed for microglial cells, curcumin counteracted the detrimental effects of TNF-α on OP differentiation and mitochondrial function, protecting cells from inflammatory damage [260]. The compound also enhanced mitochondrial integrity and function, further supporting oligodendrocyte maturation and counteracting apoptosis by endoplasmic reticulum protection as was found by Yu et al. (2012) [261]. Seady et al. (2025) revealed a similar action of curcumin on astrocytes, via NF-κB and TLR4 signalling. Their studies about the protective effect of curcumin in LPS-induced S100b secretion by astrocytes sustained a large body of evidence regarding the protective effects of curcumin on glial cell populations. Through these mechanisms, curcumin exerted neuroprotective effects, offering potential therapeutic benefits also for demyelinating diseases [262].

Addressing these knowledge gaps will require further studies and literature reviews to elucidate curcumin’s full potential and mechanisms of action in cerebrovascular health.

## Figures and Tables

**Table 1:** Curcumin’s neuroprotective effects in neurodegenerative diseases stated as in mechanisms, disease-specific impacts, and beneficial outcomes.

	Mechanism/Effect	Physiological Effects
Anti-inflammatory	Inhibits pro-inflammatory cytokines (TNF-α, IL-1β) and NF-κB signalling.	Reduces chronic neuroinflammation, a key factor in AD
Antioxidant	Scavenges free radicals, enhances glutathione, SOD, and catalase activity	Protects neurons from oxidative damage in PD
Amyloid aggregation inhibition	Binds to Aβ, preventing their formation and facilitating disaggregation	Mitigates the hallmark pathology of AD
Mitochondrial protection	Preserves mitochondrial membrane integrity, reduces oxidative stress within mitochondria	Prevents energy deficits and apoptotic signalling, critical in PD
Metal chelation	Binds metals like iron and copper, reducing metal-induced oxidative damage	Decreases oxidative stress in AD and PD
Autophagy modulation	Enhances autophagic processes, promoting clearance of damaged proteins	Prevents accumulation of toxic aggregates in diseases like AD and PD
Neurogenesis stimulation	Increases BDNF expression	Supports synaptic plasticity and neuronal survival in various neurodegenerative contexts
Apoptosis inhibition	Downregulates caspase-3 expression	Prevents neuronal loss in diseases such as PD

**Table 2:** Synthetic overview of curcumin molecular and cellular actions in cerebral and cerebrovascular diseases.

Curcumin Effects in Cerebral and Cerebrovascular Homeostasis
reduced neuronal death and promoting the microglial activation in ischemic stroke [95,96,97]
increased Iba1 microglial activation in haemorrhagic and ischemic stroke [210,211,212]
inhibition of the Cas-3, Fas/FasL, Bax and Bcl2 [72,73]
proteasome protection [77,78]
increasing proteasomal degradation of Aβ aggregates [142,143,144]
increasing the mRNA and protein for TrkB and BDNF [84]
reduction in IL-1β in haemorrhagic stroke [61]
reducing the NMDA-mediated excitotoxicity in stroke [82,83]
decreasing the VEGF expression [129,130]
inhibiting matrix metalloproteinases (MMP2, MMP9) [98]
regulating DA receptor expression and signalling [218]
downregulation of phospho-mTOR and phosphor-p70S6K [201,202,203]
NQO1 inhibition, Nrf2 activation, increasing mRNA, and catalytic activity of the CAT, SOD, and GPx1 [62,63,64,66,67,68]
free radical scavenging [49,50,51]
MCAP-1 and TNF-α inhibition in stroke [60]
reducing the oxidative stress-induced mitochondrial dysfunction [9,21,57]
counteracting the MPTP-induced dopaminergic lesion, increasing the DA levels in the frontal cortex and striatum; and inhibiting the MAO [160,161]
spatial learning and memory improvement [175]
total glutathione levels increasing [179,182]
decreased the sorbitol level by inhibition of the aldose-reductase activity [131]
reducing the myoinositol formation in endothelial and glial cells [131]
inhibition of the AGEs synthesis in endothelial cells [213]
decreasing the cell swelling by a general inhibition of the polyol pathway [226]
reducing apoptosis via JNK, ERK, and PKA [9,21,57,68,77,78,95,97,128,153,229]
reducing BBB leakage [129,130]
decreasing α-syn aggregation [210]
preventing the neurofibrillary tangle formation [141,145]
promoting nerve regeneration by NGF upregulation [230,231]
increasing NO bioavailability by eNOS protection [125,232]
decreasing iNOS activity [200,201,202,214]
reducing heavy metals-induced neurotoxicity [246,247,251]

Notations: Iba1, Cas-3, caspase 3, Fas and Fas ligand (FasL), a type II membrane protein that is part of the TNF superfamily, TrkB, tropomyosin-related kinase receptor type B, BDNF, brain-derived neurotrophic factor, IL-1β, interleukin 1β, VEGF, vascular endothelial growth factor, MMP 2/9, matrix metalloproteinase 2 or 9, NQO1, NAD(P)H: quinone oxidoreductase 1, MCAP-1, monocyte chemoattractant protein-1, TNF-α, tumour necrosis factor α, AGEs, advanced glycated endo-products, NGF, neuronal growth factor, eNOS, endothelial nitric oxide synthase, iNOS, inducible nitric oxide synthase.

## List of Abbreviations

6-OHDA	6-hydroxydopamine
AD	Alzheimer’s Disease
AGEs	advanced glycated endo-products
Aβ	amyloid-beta
b.w.	body weight
BBB	blood–brain barrier
BDNF	brain-derived neurotrophic factor
Cas-3	caspase 3
CAT	catalase
CGM	curcuma-galactomannosides
COX-2	cyclooxygenase-2
DAT	dopamine transporter
DOPAC	3,4-dihydroxyphenylacetic acid
eNOS	endothelial nitric oxide synthase
GFAP+	glial fibrillary acidic protein
GPx	glutathione peroxidase
GSH	glutathione
GSK-3β	glycogen synthase kinase-3β
i.m.	intramuscular
i.p.	intraperitoneal
Iba1+	ionised calcium-binding adaptor molecule 1
IᴋB	inhibitor of NF-ᴋB
IL-1β	interleukin-1 beta
IL-6	interleukin-6
IRF	interferon regulatory factors
LDL	low-density lipoproteins
LPS	lipopolysaccharides
MAO	monoamine oxidase
MAO-B-	monoamine oxidase B
MAPK-	mitogen-activated protein kinase
MCAP-1-	monocyte chemoattractant protein-1
MDA-	malondialdehyde
MMP-9-	matrix metalloproteinase 9
MPDP+	1-methyl -4-phenyl-2,3-dihydropyridine
MPP+	1-methyl-4-phenylpyridine
MPTP	1-methyl-4-phenyl-1,2,3,6-tetrahydropyridine
MTHFR	methylene tetrahydrofolate reductase
NDDs	neurodevelopmental diseases
NF-ᴋB	nuclear factor kappa-light-chain-enhancer of activated B cells
NFTs	neurofibrillary tangles
NLRP3	NOD-like receptor pyrin domain-containing 3
NMDA-N	methyl-D-aspartate
NO	nitric oxide
NQO1	quinone oxidoreductase 1
Nrf2	nuclear factor-erythroid 2-related factor 2
P50 p65/c-Rel	family of NF-ᴋB dimers
PAMAM	encapsulation in surface-modified polyamidoamine
PD	Parkinson’s Disease
PI3K	phosphatidylinositol 3-kinase
PINK1	phosphatase and tensin homolog-induced kinase 1
PPAR-γ	peroxisome proliferator-activated receptor-γ
Px	peroxidase
RAGE	receptor for AGE
RNS	reactive nitrogen species
ROS	reactive oxygen species
SN	substantia nigra
SOD	superoxide dismutase
SREBPs	sterol regulatory element-binding proteins
TC	tetrahydrocurcumin
TGFβ	transforming growth factor-β
TH	tyrosine hydroxylase
TNF-α	tumour necrosis factor-α
TrkB	tropomyosin-related kinase receptor type B
VEGF	vascular endothelial growth factor
